# 

**Published:** 1890-03-08

**Authors:** 


### NEW OFFICES IN THE STRAND.

The Hospital *is now published at* **140, Strand, W.C.** *It has been found desirable to establish* The Hospital *in this central and convenient locality, owing to the rapid extension of the business of this paper of late.*

### NOTICE TO CORRESPONDENTS.

All MS., Letters, Books for Review, and other matters intended for the Editor should be addressed The Editor, The Lodge, Porchester Square, London, W.

**Notice to Advertisers and Subscribers.**

All Advertisements, Orders for Copies of The Hospital, and Business Communications should be addressed to The Publisher, *not to the Editor*, The Hospital, 140, Strand, W.C.

**Bound Volumes of "The Hospital."**

Vol. VI., containing full page portraits of T.R.H. the Prince and Princess of Wales, and Miss Florence Nightingale, is now ready, 4s. post free. Cases for binding may be had of the Publisher, at 1s. 6d. each.

**To Residents, Secretaries, and Matrons.**

The Editor will be glad to have the Regulations and each Report as issued by Asylums, Hospitals, Convalescent and Nursing Institutions, as well as those of other Charities, and an early copy in duplicate of all Appeals and Festival Notices, etc., addressed to *The Editor, The Lodge, Porchester Square, London, W.* Any superintendent, secretary, matron, or other chief official who regularly sends such information may be registered, on application to the Publisher, to receive free of cost a cover for binding The Hospital in half-yearly volumes.

### SCRAPS AND GLEANINGS.

THE annual general meeting of the Royal Westminster Ophthalmic Hospital was held on Thursday last.

OSWALD A. BROWNE, M.A., M.R.C.P., has been appointed Assistant Physician to the Metropolitan Hospital.

THE Church of England Scripture Readers' Association is appealing for donations to raise a much needed £1,000.

DR. JAMES BELL was on Saturday re-elected president of the Institute of Chemistry of Great Britain and Ireland.

THE Jews' Hospital and Orphan Asylum at Norwood sheltered 267 children last year. It is very economically managed.

ERNEST H. BROCK, M.B., M.R.C.S., has been appointed Resident Medical Officer to the Royal Chest Hospital, City Road.

THE annual meeting of the American Medical Association will be held this year at Nashville, on May 20th and following days.

A DINNER in aid of the Royal Medical Benevolent College will be held at the Hotel Metropole on April 17, Sir James Paget in the chair.

INFLUENZA blows some people good. Dr. Tobin, of Waterford, has earned 12 guineas by acting as *locum tenens* for his stricken colleagues.

THE Eccles and Patricroft Hospital treated 1,004 out-patients and 116 in-patients last year—a great increase on the numbers of the previous year.

A SCHOLARSHIP has been founded in memory of Miss Helen Prideaux, who died of diphtheria while acting as medical officer to a children's hospital.

THE death is announced, at Norton, near Taunton, of Joseph Shattock, aged 102 years. There is living in Taunton a man named William Adlam who is in his 105th year.

THE Council of the Sanitary Institute have accepted an invitation from the Town Council of Brighton to hold the autumn congress and health exhibition in that town in September next.

THE Bishop of the Diocese presided at the annual meeting of the Alexandra Hospital for Children, Brighton. The report was satisfactory, and a balance was carried over to next year.

TOWARDS the proposed fund of £40,000 for erecting new buildings for the medical department, library, and examination hall at the Yorkshire College, Leeds, the Duke of Devonshire has subscribed £250.

AN epidemic similar to influenza is spreading rapidly in Bombay. Large numbers of children are unable to attend school, and work at some of the mills is almost stopped from the illness of the employés.

WESTMINSTER MEMORIAL COTTAGE HOSPITAL, SHAFTESBURY.—There were 10 patients in hospital at the beginning of the year, and 56 new cases were admitted during the year, making a total of 66 patients treated.

AN old pensioner, named James McKenna, who had taken part in the battle of Waterloo, died at Newry last week, aged 109 years. He enjoyed good health to the last, and was able to read without the use of spectacles.

THREE of the medical staff of the Stockport Provident Dispensary have sent in their resignations because the fourth member of the staff is said to have a dispensary of his own, where he gives advice at fees below the recognised standard.

DR. PARKER, preaching in favour of the London Temperance Hospital, said they knew that on that hospital there rested no stain or stigma of sectarianism. He would not ask a penny for any hospital that had its ecclesiastical and invidious distinctions and classifications.

MR. ASTOR'S will has been published. He leaves 110,000 dols. to St. Luke's Hospital and the same amount to the Cancer Hospital in New York, 400,000 dols. to the Astor Library, and a sum of 160,000 dols. is devoted to legacies to friends, charities, and various artistic and literary objects.

QUEEN CHRISTINA has given the Grand Cross of the Order of Charles III. to Drs. Candela and Sedesma, who attended on her son during his illness, and by way of showing her gratitude the Queen presented to these physicians the Insignia, Crosses, and Stars of this Order mounted in brilliants.

THE Croydon County Council have passed a resolution declaring that it would be a great advantage if the Local Government Board would procure power for sanitary authorities to require that none but duly qualified and registered plumbers should be engaged in carrying out sanitary work.

THE resolution proposed by some members of the honorary active medical staff at the annual meeting of the German Hospital, Dalston, viz., to delegate as members of the committee two gentlemen elected from their number within a fortnight from the holding of the general court, has been rejected on a poll by 2,879 votes against 1,040.

THE Johnson Hospital has come in for a handsome contribution to its endowment fund. Mr. Algernon Peckover, of Wisbech, who was joint executor with the late Canon Moore, under the will of the late Miss Charinton, of Spalding, and who was one of the residuary legatees, has presented £1,000 to the above fund.

THE Association of Public Sanitary Inspectors of Great Britain on Saturday adopted the report of the council, stating that they consider the conditions under which sanitary inspectors are appointed and discharge their duties are inimical to the public health, and are due to the defective state of the laws on sanitary matters.

THE Village Hospital for Buckhurst Hill, Chigwell, Chingford, Loughton, and Woodford, did most successful work during the past year. 62 patients were received, 32 cured, and 16 relieved. The committee are glad to say they have still retained the services of Nurse Ventum, and have raised her salary in appreciation of her valuable work.

THE Cottage Hospital at Calverton End had a deficit last year amounted to £21 3s. 1½d., and this has unfortunately increased to £32 7s. 0½d., mainly accounted for by the necessary repairs and alterations of the interior of the building and laying on the water for the waterworks supply; 27 patients were 505 days in the hospital, against 37 patients 762 days in previous year.

